# Une tumeur rare de la paroi thoracique: le synovialosarcome

**DOI:** 10.4314/pamj.v9i1.71174

**Published:** 2011-05-09

**Authors:** Yassine Ouadnouni, Mohamed Smahi, Mohammed Bouchikh, Abdellah Achir, Yassine Msougar, Marouane Lakranbi, Abdelatif Benosman

**Affiliations:** 1 Service de chirurgie thoracique, Hôpital Ibn Sina Rabat, Maroc

**Keywords:** Chirurgie, synovialosarcome, Thorax, Maroc

## Abstract

Les sarcomes ténosynoviaux ou synovialosarcomes sont des tumeurs malignes des tissus mous, l'atteinte primitive de la paroi thoracique est rare. Nous rapportons l'observation d'un patient suivi pour des douleurs thoraciques avec à l'examen une masse pariétale, le bilan radiologique a montré un développement également endothoracique de la tumeur. L'analyse anatomopathologique de la biopsie de la masse a conclu à un synovialosarcome, le patient a eu une résection de la tumeur associé à une radio chimiothérapie, le suivi a été marqué par la récidive au bout d'un an. A travers cette observation, nous insistons sur la rareté de la localisation, les caractères histologiques, les différentes approches thérapeutiques et le pronostic de ces tumeurs qui reste péjoratif.

## Introduction

Le synovialosarcome est une tumeur rare des tissus mous, de haut grade de malignité, qui atteint principalement les membres à proximité des grosses articulations. Le synovialosarcome primitif de la paroi thoracique est extrêmement rare. Nous rapportons l′observation d′un patient suivi dans notre formation pour des douleurs thoraciques en rapport avec une masse pariétale étiquetée synovialosarcome.

## Observation

**Figure 1:**
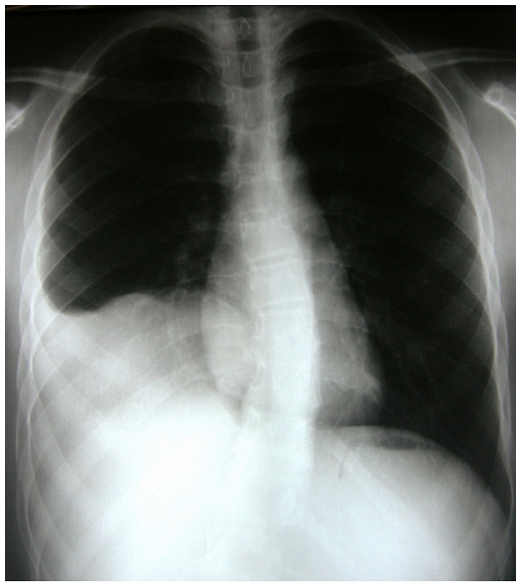
Radiographie thoracique de face montrant une opacité de tonalité hydrique basithoracique droite avec une ligne bordante évoquant une pleurésie.

**Figure 2:**
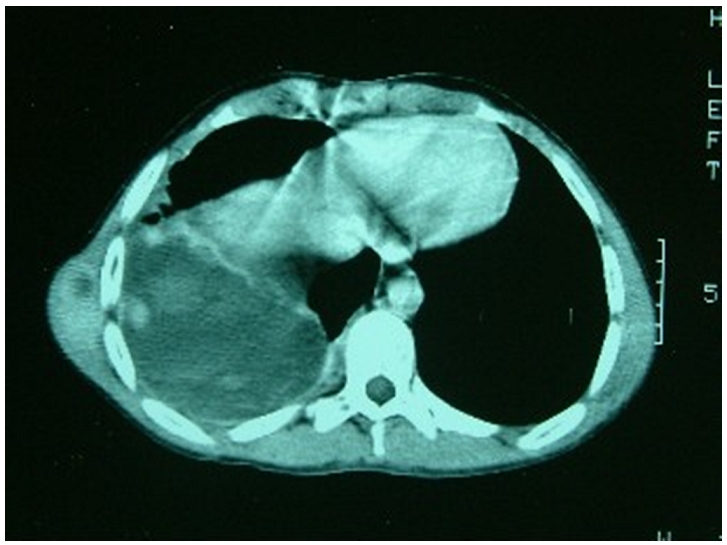
Tomodensitométrie thoracique fenêtre médiastinale objectivant un processus tissulaire à développement endo et exo thoracique avec pleurésie enkysté.

Monsieur D.K est âgé de 18 ans, sans antécédents pathologiques notables, hospitalisé dans le service pour une masse pariétale thoracique. Le patient se plaint depuis 8 mois de douleur basi-thoracique droite avec l′apparition d′une masse thoracique augmentant progressivement de volume. L′examen a trouvé une tuméfaction axillaire basse droite de 6/5 cm, ferme, fixe, peu douloureuse; aucune autre lésion n′a été objectivée, l′état général a été conservé. La radiographie thoracique a montré une opacité basale droite de tonalité hydrique (Figure 1). La tomodensitométrie thoracique a confirmé la présence d′une masse ovoïde de 5x5x2 cm aux dépend du muscle grand dentelé droit, se rehaussant après injection de produit de contraste avec une composante nécrotique. Cette masse tumorale présente une extension endothoracique en sablier à travers l′espace intercostal avec une composante charnue de même aspect faisant 4x2 cm, sans lyse osseuse; s′accompagnant d′un important épanchement pleural multicloisonné (Figure 2). L′échographie abdominale a été normale ainsi que la fibroscopie bronchique. Une ponction biopsie de la masse a conclu à un synovialosarcome. Une intervention chirurgicale a été proposée, on a réalisé une thoracotomie postéro latérale passant par le 6éme espace intercostal droit avec une incision cutanée à cheval sur la tumeur, cette dernière est en grande partie kystique et en bissac dont l′orifice de communication se trouve à hauteur de la jonction de l′arc moyen et antérieur de la 7 ^éme^ côte et du 7 ^éme^ espace intercostal, les limites endothoraciques de la tumeur sont imprécises et elle se situe à la base du lobe inférieur qu′elle infiltre en même temps que le diaphragme.

**Figure 3:**
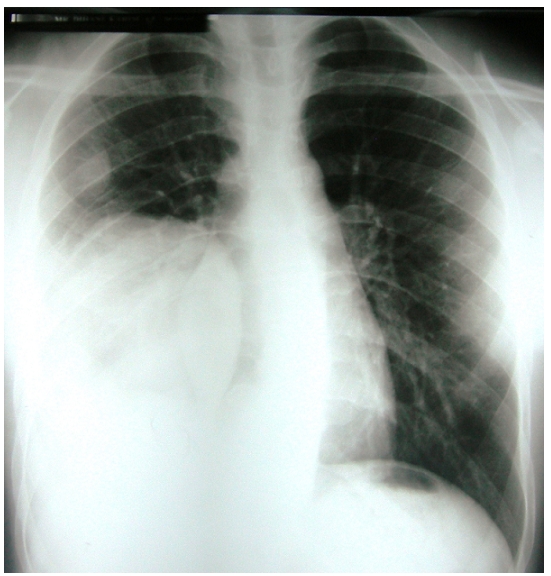
Radiographie thoracique de face montrant multiples opacités de tonalité hydrique droite.

L′exérèse de la tumeur a été réalisée en utilisant un plan extrapleural pour son décollement, néanmoins elle est palliative en raison de la nature très friable de la tumeur et de ses limites mal définies. Les suites opératoires étaient simples. L′analyse macroscopique de la pièce opératoire a trouvé une tumeur pesant 220g, en grande partie kystique avec un contenu hématique et nécrotique et par endroit un aspect blanchâtre, encéphaloïde et friable. La tumeur est constituée par une population cellulaire assez polymorphe agencée tantôt en faisceaux plus ou moins tourbillonnants entrecroisés avec parfois un aspect pseudo hémangiopéricytaire; par ailleurs, ces cellules tapissent des cavités de dimension variable prenant un aspect endothéliforme; elles montrent des atypies cytonucléaires modérées à franches, l′index mitotique est élevé évalué à 53 mitoses sur 10 champs, à l′immuno-histochimie les cellules épithéliales expriment l′antigène membrane épithélial et la cytokératine, et les cellules fusiformes expriment la vimentine. Un traitement adjuvant a été indiqué à base d'une radiothérapie à la dose de 50 Gray, centrée sur la cicatrice de la thoracotomie associée à six cures de chimiothérapie associant l'adriamycine et l'ifosfamide. Au bout d′un an de suivi l′état général de notre patient s′est altéré, avec la constatation d′une récidive de la tumeur et la survenue de métastases parenchymateuses (Figure 3). Le patient est décédé 2 mois après.

## Discussion

Le synovialosarcome est une tumeur rare de l′adulte jeune. Sa localisation principale est décrite comme péri articulaire, au sein des tendons, des bourses et des capsules; d′autres localisations primaires atypiques sans relation avec le tissu synovial sont décrites. De ce fait, la plupart des auteurs s'accordent à dire que l'origine de cette tumeur dérive d'une cellule mésenchymateuse pluripotente à différenciation synoviale [1]. Le synovialosarcome représente 5 à 10% des tumeurs des parties molles. Il existe une discrète prédominance masculine, le sex-ratio est de 1,5. Il touche préférentiellement le sujet jeune [2]. Notre malade avait moins de 20 ans. Le synovialosarcome se présente comme une masse indolore qui augmente progressivement de volume sur plusieurs mois jusqu’à l'apparition des premiers signes. Macroscopiquement, les synovialosarcomes biphasiques associent deux contingents qui sont: des cellules épithéliales cuboïdales et/ou cylindriques, dotées d′un large noyau vésiculé et nucléolé centrant un cytoplasme clair et des cellules fusiformes en plages cohésives et au cytoplasme peu représenté. La vascularisation est soit peu marquée soit de type hémangiopéricytaire. En immuno-histochimie les cellules épithéliales expriment l′antigène membrane épithélial et la cytokératine, et les cellules fusiformes expriment la vimentine. La plupart des tumeurs portent une translocation caractéristique t(X;18), qui implique les gènes SSX1 ou SSX2 du chromosome X (Xp11) et le gène SYT du chromosome 18 (18q11). Les transcrits du gène de fusion SYT–SSX peuvent être détectés sur les prélèvements anatomopathologiques avec une sensibilité de 96% et une spécificité de 100% [3].

Compte tenu de sa rareté, le synovialosarcome de la paroi thoracique est de diagnostic difficile, son caractère primitif est difficile à affirmer, la recherche d'une tumeur primitive extra thoracique est fondamentale avant de poser le diagnostic. Dans notre cas, le diagnostic a été retenu au terme d'une analyse anatomopathologique caractéristique des synovialosarcomes para articulaires. L'absence d'une localisation tumorale extra thoracique au moment du diagnostic et après 18 mois de recul confirme le caractère primitif de la tumeur. La radiographie thoracique révèle une opacité de tonalité hydrique homogène bien limitée, les calcifications sont retrouvées dans plus de 25% des cas. Dans 20 % des cas, l'os adjacent est atteint, soit sous la forme d'une réponse à la pression avec ostéosclérose, soit sous la forme d'un envahissement osseux avec érosions corticales. La tomodensitométrie permet de mieux apprécier la présence des microcalcifications, de préciser l'extension endo et exothoracique de la tumeur ainsi que les signes de malignités (aspect hétérogène avec nécrose centrale, pleurésie, invasion médiastino-pulmonaire). A l′imagerie par résonance magnétique, environ 90 % des synovialosarcomes sont bien limités avec parfois un aspect de capsule, la présence de lobulations ou de cloisons est fréquente. Dans 80 % des cas, les tumeurs sont hétérogènes en T2 avec des signaux de tonalité liquidienne, solide, ou fibreuse [4]. Le synovialosarcome est connu pour son caractère agressif et son risque élevé de métastases. Le traitement, comme dans l'ensemble des sarcomes des parties molles, est multimodal associant la chirurgie et la radio-chimiothérapie. La chirurgie d'exérèse isolée donne des résultats aussi bons qu'une association à la radio chimiothérapie. Cependant le traitement multidisciplinaire permet une chirurgie d'exérèse moins mutilante avec un meilleur contrôle tumoral local et des métastases à distances. Les facteurs de mauvais pronostic sont: la taille de la tumeur initiale de plus de 5 cm de diamètre, l'invasion locale, le haut grade histologique (index mitotique, pourcentage de nécrose) et la résection chirurgicale incomplète. L'exérèse chirurgicale complète de la tumeur, lorsque qu'elle est possible fonctionnellement et techniquement, reste le facteur déterminant pour la survie à long terme [4].

## Conclusion

Les sarcomes ténosynoviaux ou synovialosarcomes sont des tumeurs malignes des tissus mous, dont la localisation primitive thoracique est rare. Son traitement, est avant tout chirurgical comme dans l'ensemble des sarcomes des parties molles, associant une radiothérapie pour un meilleur contrôle local, toutefois, son pronostic reste sombre devant une tumeur de plus de 5 cm de diamètre, une invasion locale, un haut grade histologique et une chirurgie incomplète.
